# Building on the Ccr4‐Not architecture

**DOI:** 10.1002/bies.201600051

**Published:** 2016-08-22

**Authors:** Zoltan Villanyi, Martine A. Collart

**Affiliations:** ^1^ Faculty of MedicineDepartment of Microbiology and Molecular MedicineUniversity of GenevaGenevaSwitzerland; ^2^ Institute of Genetics and Genomics GenevaGenevaSwitzerland

**Keywords:** architecture, Ccr4‐Not complex, deadenylation, mRNA circularization, mRNA decay, protein folding, subunit localization

## Abstract

In a recent issue of Nature Communications Ukleja and co‐workers reported a cryo‐EM 3D reconstruction of the Ccr4‐Not complex from *Schizosaccharomyces pombe* with an immunolocalization of the different subunits. The newly gained architectural knowledge provides cues to apprehend the functional diversity of this major eukaryotic regulator. Indeed, in the cytoplasm alone, Ccr4‐Not regulates translational repression, decapping and deadenylation, and the Not module additionally plays a positive role in translation. The spatial distribution of the subunits within the structure is compatible with a model proposing that the Ccr4‐Not complex interacts with the 5′ and 3′ ends of target mRNAs, allowing different functional modules of the complex to act at different stages of the translation process, possibly within a circular constellation of the mRNA. This work opens new avenues, and reveals important gaps in our understanding regarding structure and mode of function of the Ccr4‐Not complex that need to be addressed in the future.

## Introduction

Ccr4‐Not is a conserved multi‐subunit, multifunctional eukaryotic regulator. It has been proposed to integrate environmental signals and coordinate multiple nuclear and cytoplasmic steps in gene expression (reviewed in 1, 2, 3, 4, 5, 6, 7). The core Ccr4‐Not complex is composed of six conserved subunits, Not1, Not2, Not5, Caf40, Ccr4, and Caf1. Not4 is a core subunit in yeast, and it is conserved in most eukaryotes, but its association with the core in human and flies is less tight 8. Trypanosomes are an exception since they lack both Not4 and Ccr4 9. They express a minimal complex providing some basic functions that are sufficient in this species probably because of differences in mechanisms of gene regulation, as will be discussed below. There are additional species‐specific subunits, such as Caf130 in budding yeast, a close to stoichiometric subunit Mmi1 in fission yeast, or Not10 and Not11 in human, flies and trypanosomes. The complex is organized in functional modules that dock onto the large Not1 subunit that serves as a scaffold. Not1 tethers the other Ccr4‐Not subunits to messenger RNA (mRNA) substrates, in particular its enzymes: the Caf1 and Ccr4 deadenylases and the Not4 E3 ubiquitin ligase. Not1 itself is recruited directly or indirectly to mRNAs by multiple different tethers, including RNA binding proteins and the micro RNA machinery. The translational repressor and decapping activator, called Dhh1 in yeast and DDX6 in human, also docks onto the Not1 scaffold.

Many different reviews about the Ccr4‐Not complex were published in the last few years 1, 3, 4, 5, 7, 10, 11, 12, 13, 14, 15, 16, 17, 18, 19 summarizing the diversity of functions identified for this complex and underlying how it contributes to regulate gene expression at all steps, from production of mRNAs in the nucleus to their degradation in the cytoplasm. In addition, it was shown recently that in the nucleus Not1 is imprinting ribosomal protein (RP) mRNAs during transcription in a Not5‐dependent manner and that this mRNA imprinting is facilitating RP translation in the cytoplasm 20.

Thus, Ccr4‐Not physically connects processes in the nucleus during production of mRNAs and in the cytoplasm during decay of mRNAs, and in addition it plays dual opposing roles in translation. Knowing the overall architecture of the Ccr4‐Not complex can give cues about the distribution of available surfaces around the assembly for regulation of gene expression processes in these diverse ways. It should provide mechanistic explanations for why a single regulator has evolved to act at all of these different steps.

Within the last 5 years several beautiful high‐resolution X‐ray structures for the conserved functional subunits and modules have become available (see review 21). This has been very useful to clarify the mechanisms of individual functions of the Ccr4‐Not complex. For instance, the specificity of Ccr4 for poly(A) substrates has been understood through the high resolution structure of the nuclease domain of a human Ccr4 homolog 22. Similarly a high resolution structure of the RING domain of Not4 in complex with Ubc4 has revealed that the specificity of Not4 for Ubc4 can be explained by the more extensive interaction interface possible between Not4 and this specific E2 enzyme 8. The atomic model of Caf40 showing its structural similarity to Armadillo‐repeat proteins indicated that it provides a platform for protein‐protein interactions, while the shape and electrostatic potential of its surface is consistent with nucleic acid binding properties 23. It was also discovered that the catalytic sites of Ccr4 and Caf1 are largely accessible within an assembled nuclease module consisting of Ccr4, Caf1, and a central domain of Not1 24. Moreover through the resolution of a complex between this same central domain of human Not1 (CNOT1) and DDX6 it was demonstrated that the binding of the nuclease subunits and DDX6 can occur simultaneously because it takes place on opposite sides of Not1 25, 26. An open question concerning how the microRNA machinery was recruiting the Ccr4‐Not complex to mRNAs was solved by the resolution of the complex of CNOT9 (orthologous to Caf40) with CNOT1 revealing tryptophan‐binding pockets in CNOT9 25, 26. Finally high resolution structures of the NOT module consisting of the C‐terminal domain of Not1 and the Not2–Not5 heterodimer 27, 28, as well as the ubiquitination module consisting of this same C‐terminal domain of Not1 in complex with Not4, clarified that Not4 and the Not2–Not5 heterodimer bind independently. It also revealed that the NOT module forms a composite surface with a tool like V‐shape, likely to serve as a versatile platform for macromolecular interactions. Moreover, patches of positively charged residues within the Not module are probably responsible for its ability to bind poly(U) RNA in vitro 27, 28.

Until the new EM structure provided by the work of Ukleja et al. published this year in which the different subunits were also immunolocalized 29, our only information about the overall landscape of the Ccr4‐Not complex was a low resolution (30 Å) EM structure from *Saccharomyces cerevisae* 30. We will discuss here the impact that this new information about the architecture of the complex has on our understanding of this complex from a functional point of view, and also present in brief how the data was obtained, its limitations and perspectives.

## Understanding the architecture of Ccr4‐Not

**Figure 1:**
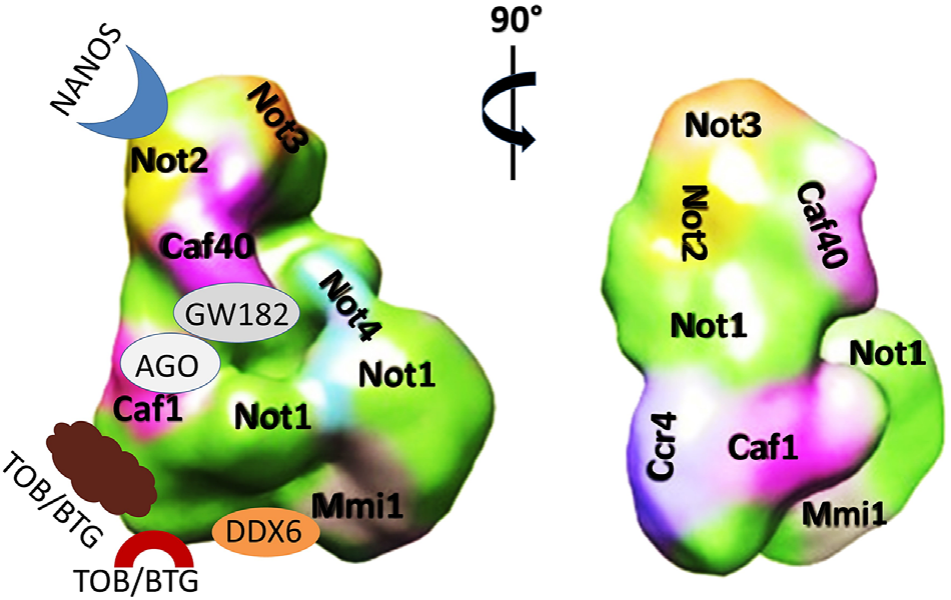
Ccr4‐Not complex of *S. pombe* with and without a projection of docking sites for various eukaryotic interaction partners. This figure was adapted from 29. It shows two orthogonal views of the Ccr4‐Not complex with (left) or without its interaction partners (right). Left, binding sites of the known eukaryotic Ccr4‐Not‐interacting partners: Tob/BTG (that binds to Caf1) and Nanos, TTP and DDX6 (that bind to different parts of Not1), the GW182 is also shown as it is an essential component of miRNA‐targeted recruitment of the Ccr4‐Not complex to mRNA.

Ukleja et al. applied electron microscopy (EM) combined with medium resolution 3D information and docking of existing high‐resolution structures to generate a pseudo‐atomic model of the Ccr4‐Not complex of *Schizosaccharomyces pombe* 29. By combining immuno‐microscopy and RNA‐nanogold labeling techniques they provided for the first time a comprehensive molecular model of the full Ccr4‐Not complex. They docked the experimentally determined or existing model structures into the electron density map of the complex and then applied a hybrid modeling approach to complete their architectural definition of the complex, by simultaneous docking of structures into the density map using spatial restraints, and additionally modeling disordered and flexible regions (Fig. 1).

To purify the complex, the authors made use of the TAP (tandem affinity purification) tag fused to the Not2 subunit at its C‐terminus to purify the complex by IgG sepharose‐binding and TEV cleavage‐mediated elution followed by separation on a glycerol gradient. High‐salt conditions were used to avoid co‐purification of non‐specific components. For cryo‐EM the authors applied low glutaraldehyde concentrations in the glycerol gradient to provide stabilization of the macromolecular structures (GraFix) 31. Immuno‐EM was then used to localize each GFP‐tagged subunit within the 3D reconstruction of the Ccr4‐Not complex purified by Tap‐tagged Not2.

Besides conserved Ccr4‐Not subunits, an additional essential fission yeast‐specific protein called Mmi1 was co‐purified in close to stoichiometric amounts. By retaining Mmi1 on the IgG sepharose beads while eluting the Ccr4‐Not complex, the authors could define the position of Mmi1 by the change in volume of the Ccr4‐Not complex. They confirmed Mmi1 location in the complete structure using negative staining EM of an RNA oligonucleotide with four tandem motifs recognized by Mmi1 fused to Nanogold. The presence of Mmi1 as a close to stoichiometric subunit in the fission yeast Ccr4‐Not complex was a surprise. Mmi1 is a nuclear RNA binding protein that is important for removal of meiosis‐specific transcripts during vegetative growth 32, 33 and it also plays critical roles in heterochromatin integrity 34. Its close to stoichiometric presence in the purified fission yeast Ccr4‐Not complex suggests a critical role of Ccr4‐Not in meiosis and/or heterochromatin formation in *S. pombe*. Indeed, a recent study reported that Mmi1 recruited the Ccr4‐Not complex to its RNA targets. Curiously Ccr4 and Caf1 have no effect on stability of these Mmi1 targets. Instead they have an impact on heterochromatin integrity, as does Not4 35.

## Integrating architecture with function

Ukleja et al. analyzed the entire Ccr4‐Not architecture in light of the importance of the Ccr4‐Not complex for repressing gene expression at the level of translation repression 36, 37, 38, decapping 39, 40, and mRNA deadenylation 41, 42, 43. They observed that the site of the complex onto which DDX6 docks is located in close proximity to the region where Ccr4 and Caf1 are situated, but is on a different face of the complex (Fig. 1). Hence they proposed a model in which DDX6 bound to the Ccr4‐Not complex interacts with the 5′ end of an mRNA, while Ccr4 and Caf1 are connected to the 3′ end of the same mRNA such that the 5′ and 3′ ends of the mRNA targeted for repression will be spatially brought together. Such a model is compatible with previous findings reporting that 5′ and 3′ ends of mRNAs are located spatially in close proximity, with the finding that deadenylation and decapping can occur at the same time in polysomes 44 and with the fact that both processes are regulated by Ccr4‐Not 42, 45. Interestingly, the deadenylase module is located on the same side of the Ccr4‐Not complex as the binding sites for proteins that tether the complex to mRNAs, such as the Nanos 46 and tristetraprolin 47 RNA binding proteins or the microRNA machinery 25, 48 (Fig. 1). This is consistent with a role for these proteins in tethering mRNA poly(A) 3′ ends close to the deadenylase module.

The model put forth by Ukleja et al. shows mRNAs to be repressed exclusively engaged with one side of the Ccr4‐Not complex. This raises the intriguing question of what role the other side of the Ccr4‐Not complex may play.

## Ccr4‐Not within a circular configuration of mRNAs during translation

**Figure 2:**
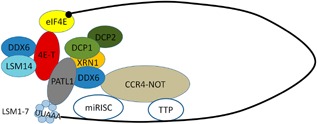
Interaction of Ccr4‐Not with the decapping and mRNA decay machinery. This figure was adapted from 50. The mRNA decapping and decay machinery including the Ccr4‐Not complex are organized in a circular constellation.

Former studies have proposed a mode of translation in which mRNAs are organized in a circular form, stabilized through the interaction of translation initiation factors associated with the 5′ end of the mRNA and the poly(A) binding protein associated with the 3′ end of the mRNA. It was suggested that this interaction between the two ends of the mRNA facilitates translation 49. We recently described that Not5 has a selective role on Not1 association with mRNAs in the nucleus (mRNA imprinting) and that this facilitates their translation. Indeed, Not5 is needed for binding of Not1 to ribosome protein‐encoding (RP) mRNAs. In *not5Δ,* binding of Not1 to these mRNAs is reduced and instead Not1 associates with other mRNAs, whose presence in polysomes consequently increases while that of RP mRNAs decreases. 20. It is tempting to propose that this positive role of Ccr4‐Not on translation can occur within a circular mRNA constellation. Such a configuration has already been proposed during gene repression, through interactions between eIF4E‐Binding Protein, 4E‐T, and DDX6 26, 50 (Fig. 2).

**Figure 3:**
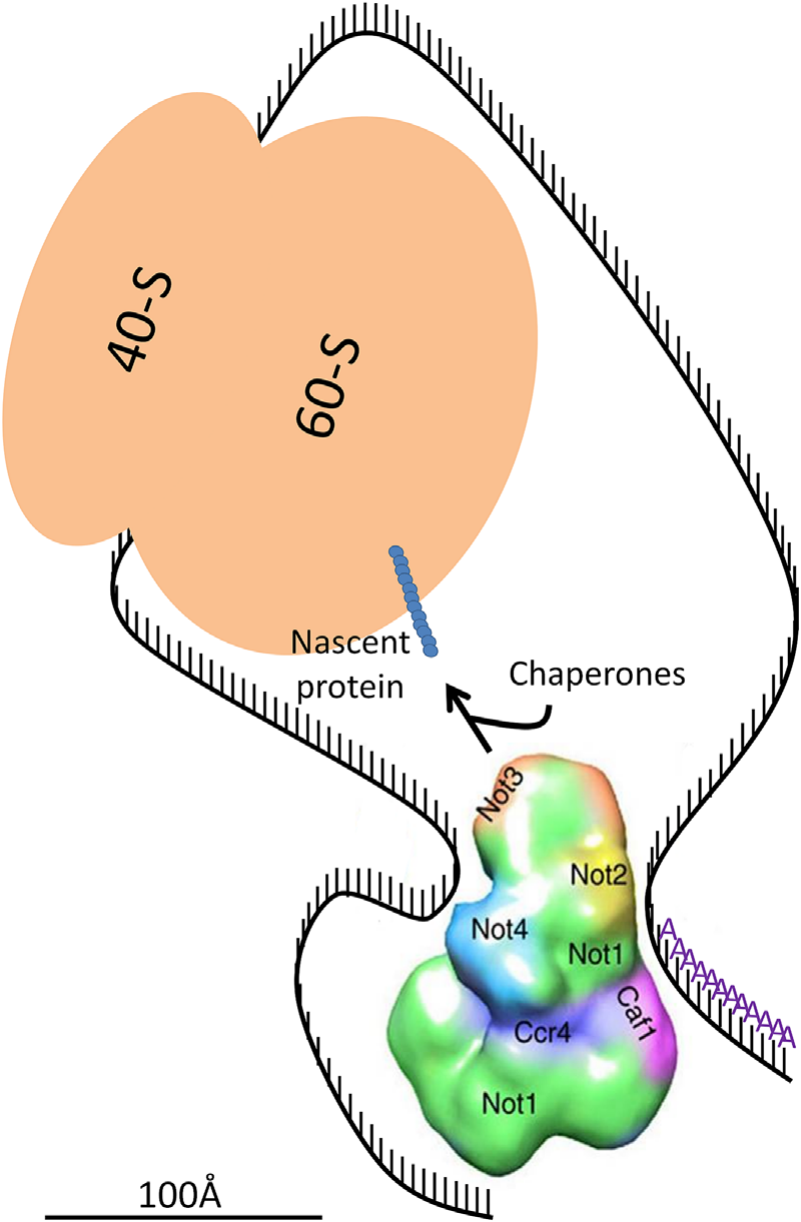
Model for the interaction between mRNA and the Ccr4‐Not complex during the translation process. The Ccr4‐Not complex structure was adapted from 29. Not4 via its RNA Recognition Motif (RRM) could tether the mRNA with a translating ribosome in close proximity to the Ccr4‐Not complex, in particular to Not5, such that it can help chaperone or interacting partner association with the nascent polypeptide chain and more generally allow the Not proteins to participate in co‐translational events. As translation elongation proceeds, the 5′ end of the mRNA can become accessible to the site where DDX6 docks onto the Ccr4‐Not complex. The 3′ end of the mRNA instead is accessible to the other side of the Ccr4‐Not complex, where it can be tethered by RNA binding proteins (not depicted), such that the poly(A) sequences are accessible to the Ccr4 and Caf1 nucleases. Size proportion of the shapes representing macromolecules are similar to the real size proportion of the represented macromolecules. The mRNA depicted is 160 nucleotides in length. Scale bar ≈ 100 Å.

Several studies have indicated that translation and mRNA decay are physically connected 44, 51. In one recent work it was shown that co‐translational 5′ to 3′ mRNA decay is widespread with 5′ to 3′ decay following the translating ribosome 52. Taking all of the above into consideration, one can assume that a regulator playing a role in both processes is the best candidate for this physical connection. This ensures a tight communication between the two processes that nevertheless should be spatially separated. Thus, we can propose an alternative constellation of interactions between mRNAs and the Ccr4‐Not complex (Fig. 3). In our model, the 5′ end of mRNAs, as the ribosome starts moving along the mRNA during translation, would be located on the Not side of the complex. Not4 has an RRM motif, and this might help to tether the 5′ region of the mRNA initially on its side of the Ccr4‐Not complex. This would place Not5 (called Not3 in *S. pombe*) facing towards the emerging nascent chain to promote co‐translational protein interactions 53. As the ribosome progresses along the mRNA, the 5′ end would gradually reach or become available to the site of the complex where DDX6 docks 26. At this point on, DDX6 would be able to contribute to mRNA translational repression or decapping, while the nuclease module would be able to deadenylate the mRNA, under conditions that require gene repression. Interestingly, in yeast Dhh1 cross‐links throughout mRNAs 54. Its presence during translation might permit a rapid recruitment at any time to the 5′ end of the mRNA to initiate decapping, by docking onto the appropriate site of the Not1 scaffold also present during translation, if repression is needed. Within such a model, recruitment of decapping factors or activation of deadenylation could be immediately effective, depending upon the Ccr4‐Not complex sensing a nascent protein folding difficulty, or perceiving an external signal, via its Not4 or Not5 subunits. One can imagine that a switch could be induced by a conformational change.

This is obviously a provocative model that is nevertheless supported by the available data and the new architecture proposed by Ukleja et al., and it can be tested experimentally. For instance, for a model such as this, one would predict that mRNAs with ribosomes stalled close to the start site of translation may be less efficiently degraded by Ccr4‐Not dependent mechanisms compared to mRNAs with ribosomes stalled further away. Ribosome profiling data has already indicated that ribosome footprints close to the beginning of ORFs are globally over‐represented 55. This so‐called 5′ ramp has been extensively discussed and analyzed. It was initially attributed to preferential use of codons corresponding to low‐abundance cognate tRNAs at the 5′ ends of genes 56, but alternative explanations have recently been put forward. One study suggests that it is due to steric constraints for the assembly of multiple ribosomes at the 5′ of ORFs, since this is observed mostly for ribosome footprints in monosome fractions but not in the polysomes 57. In another study, a combination of several factors was proposed to explain the 5′ ramp, such as codon usage, slower elongation during the first phase of translation or ribosome drop‐off 58. But this 5′ ramp is also compatible with less efficient degradation of mRNAs with ribosomes stalled close to the start that our model would predict. It is interesting to note that in this last study 58 it was calculated that shorter mRNAs have relatively higher translation initiation efficiencies, and it was proposed that the 5′UTR might sense the ORF length via a closed loop structure. Our recent finding that Not1 can imprint specific mRNAs in the nucleus during transcription and thereby regulate translation of the imprinted mRNA, together with these different observations, are supportive for the model that we propose in the present perspective. It also raises the possibility that our model might concern mainly mRNAs imprinted in the nucleus by Not1 binding 20. If so, then predictions can be tested by tethering Not5 out of the nucleus to loose mRNA imprinting by Not1, or on the contrary imposing transcriptional stress which increases and broadens targets of Not1 mRNA imprinting 20.

As mentioned above, trypanosomes have a minimal complex and in particular have no Not4. Hence in this organism our model is not directly applicable. Trypanosomes express polycistronic mRNAs that create numerous unwanted transcripts that are rapidly destroyed. Therefore trypanosomes are exceptional in the sense that mRNA degradation often occurs without any link to translation quality control and appears to be a major mode of gene expression regulation. Trypanosomes separated very early in evolution. It could be that the link between translation and mRNA decay implicating Not4 evolved after trypanosomes separated. Alternatively, another protein might replace Not4 that is actually not a stable subunit of the Ccr4‐Not complex in human or flies as mentioned above.

## Future challenges

The Ccr4‐Not structure proposed by Ukleja et al. is based in large part upon computational modeling that is not unambiguous. The authors verified the localization of Ccr4‐Not subunits independently, but there can be substantial errors in the orientation and exact position of the subunits. A high resolution cryo‐EM structure will be necessary to confirm this model.

In their work Ukleja et al. selected manually the highest quality single particles for best results, and smaller complex assemblies were excluded from the analysis. It might be possible to obtain a better homogeneity. Instead it is also possible that heterogeneity is a biological reality rather than an experimental limitation. Indeed, there is evidence that the Ccr4‐Not complex exists in several forms. For instance the level of some Ccr4‐Not subunits is variable between different types of tissues when the amount of the other subunits remains stable 59. In addition to the possibility that different forms of the complex exist, the difficulty to obtain crystals for X‐ray crystallography and the necessity to cross‐link for the determination of high‐resolution structural data of the entire complex, suggests that the integral complex is likely to be very flexible and/or exists in several different states. Different architectures may define different functional forms. Thus, it may be important to define the composition and architecture of all Ccr4‐Not complexes, and moreover it may be essential to purify the Ccr4‐Not complexes from different subunits because different functional forms of the complex may not have all subunits. Moreover, since the Ccr4‐Not complex is engaged in every step of the mRNA lifecycle, it is very likely that protein modifications of the external protein interaction surfaces define the functional state of the complex. If such modifications exist they might have effects on the overall structure. Finally, there are species‐specific subunits for which we still need to determine where they are placed within the Ccr4‐Not complex from these species, and how they affect the overall architecture.

## Conclusions

The work of Ukleja et al. provides us for the first time with an architectural description of the Ccr4‐Not complex. Understanding the organization of the complex and the exact locations of the different subunits within the global structure, and linking this information with existing functional knowledge, provides the first real opportunity to understand why so many functions are provided by a single entity. This allows us to come up with mechanistic models that can be experimentally tested. We propose in this perspective a possible scenario whereby the same complex can in spatially different sites, control protein production via recruitment of chaperones to a nascent peptide by the Not2/Not5 dimer and repress gene expression at both the 3′ and the 5′ ends of the mRNA by Ccr4 and Caf1 and binding of Dhh1/DDX6, respectively.

The authors have declared no conflict of interest.
